# Association between combined oral contraceptive prescription and cervical artery dissection: A retrospective cohort study

**DOI:** 10.1016/j.thromres.2025.109279

**Published:** 2025-02-07

**Authors:** Robert J. Trager, Catherine P. Haering, Anthony N. Baumann, Debbie S. Wright

**Affiliations:** a Connor Whole Health, University Hospitals Cleveland Medical Center, Cleveland, OH, USA; b Department of Family Medicine and Community Health, Case Western Reserve University School of Medicine, Cleveland, OH, USA; c Department of Biostatistics and Bioinformatics Clinical Research Training Program, Duke University School of Medicine, Durham, NC, USA; d Department of Reproductive Endocrinology and Infertility, Case Western Reserve University School of Medicine, Cleveland, OH, USA; e College of Medicine, Northeast Ohio Medical University, Rootstown, OH, USA; f Department of Rehabilitation Services, University Hospitals Cleveland Medical Center, Cleveland, OH, USA; g MSCR Student, Parker University, Dallas, TX, USA; h Private practice, The Grove Health and Wellness, Courtenay, BC, Canada

**Keywords:** Contraceptive agents, Ethinyl estradiol, Progestin, Contraceptive devices, Cardiovascular diseases, Cerebrovascular disorders

## Abstract

**Background:**

To date, research has identified positive associations between combined oral contraceptives (COCs) and adverse vascular events, however, evidence regarding the possible association with cervical artery dissection (CeAD) remains limited. We tested the hypothesis of a positive association between COCs and CeAD within one year following COC initiation compared to matched controls initiating intrauterine devices (IUDs), as measured by risk ratio (RR).

**Methods:**

We queried de-identified United States health records data (TriNetX, Inc.) from 2014 to 2024 for females aged 15–50 years without previous cerebrovascular disease or CeAD, creating mutually exclusive cohorts initiating either COCs or IUDs. We used propensity matching to control for variables associated with CeAD. Our primary outcome included the RR for CeAD within one year follow-up. We secondarily explored cumulative CeAD incidence and RR of stroke, also examining outcomes for females with ≥2 COC prescriptions (COC2).

**Results:**

After matching there were 214,020 patients per cohort (mean age 31 years). The incidence and risk of CeAD was greater among those prescribed COCs compared to matched controls with IUDs [95 % CI] (COCs: 0.016 %, IUDs: 0.008 %; RR 1.94 [1.10,3.43]; *P* = 0.0195). A similar association was observed for stroke (COCs: 0.106 %, IUDs: 0.057 %; RR = 1.86 [1.49,2.32]; *P* < 0.0001). The secondary COC2 analysis revealed similar findings.

**Conclusions:**

The present findings suggest that females prescribed COCs have an increased risk of CeAD and stroke compared to matched controls using IUDs. These observations should be viewed as preliminary, require corroboration by other studies, and in isolation do not replace the broader clinical and shared decision-making regarding contraceptive use.

## 1. Introduction

Cervical artery dissection (CeAD) is a serious vascular condition characterized by a tear in the intimal layer of the vertebral or carotid artery, leading to the formation of an intramural hematoma and potential disruption of blood flow, leading to possible stroke [1]. The incidence of CeAD is approximately 8.9 per 100,000 person-years [2], with a mean age of onset of 47 [3]. Combined oral contraceptives (COCs), also known as birth control pills, are a common form of contraceptive and contain the hormones estrogen and progestin [4]. Previous studies have identified positive associations between the use of COCs and various adverse vascular events, including ischemic strokes and venous thrombosis [5,6].

However, the association between combined COCs and CeAD has yielded inconsistent results. One case-control study identified a statistically significant positive association [7], while two other case-control studies found non-significant associations [8,9]. None of these studies were limited to females, thereby limiting the proportion of individuals who were eligible to take COCs and hindering generalizability to this demographic [7–9]. In addition, none of the studies focused exclusively on COCs as a risk factor for CeAD and did not use an active control, such as females using an alternative form of contraception [7–9]. Research focusing on the possible COC-CeAD relationship would clarify CeAD risk factors for this specific demographic, which are generally poorly understood [10].

There are several possible mechanisms whereby COCs may exert adverse vascular effects. COCs contain estrogen and progestin [4] which lead to a dose-dependent increase in platelet activity, increase in levels of clotting factors (i.e., II, VII, VIII, IX and X), fibrinogen and soluble fibrin, and decrease in antithrombin and vessel wall fibrinolytic activator [11]. Estradiol may trigger inflammation by activating macrophages [12]. Estrogen and progesterone may lead to arterial collagen degeneration [13], which could serve as a predisposing factor for arterial dissection. Finally, COCs contribute to fluid retention which could likewise increase the risk of vessel rupture [13].

Aside from COCs, intrauterine devices (IUDs) are another common contraceptive method involving insertion of a plastic device with a hormonal or copper component into the uterus [14]. Unlike COCs, IUDs have not been associated with vascular events [6,15]. Hormonal IUDs contain levonorgestrel, a progestin, which acts locally on the uterus. While a small amount of levonorgestrel is absorbed systemically, it only reaches about 4 % to 13 % of the plasma concentration compared to oral levonorgestrel present in COCs [16]. Due to their plausibly different vascular risk profiles, IUDs may serve as an optimal control when examining the potential association between COC and CeAD.

This study aims to examine the hypothesis that COC use is associated with an increased risk ratio (RR) of CeAD. We employed a retrospective cohort design, comparing the risk of CeAD in females prescribed COCs to a matched control group using IUDs. We secondarily examined the risk of stroke and repeated our analyses for females having at least two COC prescriptions.

## 2. Materials and methods

### 2.1. Study design

This study adheres to a protocol registered in Open Science Framework (https://osf.io/zmeqp) [17], with minor modifications as noted herein. We excluded females who became pregnant at any point throughout the follow-up window. The data range was shortened from 15 to 10 years (i.e., from 2014 to 2024) as the TriNetX network had grown since our initial planning of the study, enabling adequate sample size despite using a shorter time window. A more recent data range was preferred to ostensibly minimize between-cohort differences related to temporal trends for COC prescription, IUD use [18] and CeAD detection [2]. We added stroke as a positive control outcome, as COCs have been previously established as a risk factor for stroke [5,19,20]. We did not use natural language processing, a software feature which was no longer supported in our data resource. Finally, we repeated our analyses only including females prescribed at least two COCs during follow-up.

Data were sampled with a query date of October 16, 2024, using the United States (US) TriNetX network (TriNetX, Inc.; Cambridge, MA, US), a data resource including over 133 million patients attending 94 large medical centers and their affiliated offices [21,22]. TriNetX data are routinely collected and can be examined to conduct retrospective research. This data resource may be searched using standardized terminology including International Classification of Diseases, 10th Revision (ICD-10) codes, which are interconverted automatically to 9th Edition when necessary [21,22]. TriNetX monitors data quality with respect to conformance, consistency, and completeness [21,22], with medication completeness being at least 87 % [23]. A visual representation of the study design is included separately (Supplemental File 1, Fig. 1).

The health records data include information from inpatient, outpatient, and emergency care settings, providing a comprehensive view of patient interactions. These data are aggregated from electronic health records across participating institutions, and include information regarding demographics, encounters, diagnoses, procedures, medications and vaccinations, lab results, clinical findings, vital signs, genomics, and oncologic information [21,22]. TriNetX allows restriction of cohorts based on sex assigned at birth. While gender identity may be used as an additional descriptor, it was not utilized in the present study to define our cohorts.

TriNetX adheres to the Health Insurance Portability and Accountability Act (HIPAA), the US federal law that protects healthcare data privacy and security, is certified to the International Organization for Standardization 27,001:2013 standard, maintains an Information Security Management System. The University Hospitals Institutional Review Board (IRB; Cleveland, OH, US) considers studies using de-identified data from the online TriNetX platform to represent ‘Not Human Subjects Research’ thereby making the present study exempt from IRB review and waiving the need for consent.

### 2.2. Participants

#### 2.2.1. Eligibility criteria

We included females aged 15 to 50, consistent with previous studies [24], to capture females of child-bearing age. Individuals were divided into two cohorts according to either first receipt of COC prescription or first instance of IUD insertion (Supplemental File 1, Table 1). We required individuals prescribed COCs to have a prescription including both ethyl estradiol and progestin components [4]. We also required that individuals in both cohorts had any health care visit between one day and three years preceding the index date (date when receiving COC or IUD), to improve data completeness. A key aspect of this study is the use of an active control cohort, used to enhance comparability [25]. Previous strategies of examining possible cardiovascular risks of COCs included dose-response analysis or comparison with transdermal, vaginal ring, and IUD reference groups, or comparison with no hormonal contraceptive [5,6,13,24]. We selected IUDs as a comparator over other hormonal contraceptive agents due to their lack of association with vascular events [6,15].

We excluded individuals with pre-existing cerebrovascular disease, CeAD, and recent (i.e., ≤ 2 weeks) external causes of morbidity (e.g., motor vehicle collision), spinal surgery, critical care services, inpatient care, emergency visits (e.g., individuals who may have greater health complexity or pre-existing CeAD), those who were pregnant, and males (Supplemental File 1, Table 2). Exclusion of pregnancy throughout baseline and follow-up aimed to minimize confounding related to pregnancy that might otherwise develop with discontinuation of COCs or IUDs, and was necessary considering pregnancy is a known risk factor for CeAD [26,27]. To limit concurrent use of multiple hormonal contraceptive methods, we excluded those currently prescribed or using (within 3 months preceding and including index date [5]) topical, injectable, vaginal, or drug-implant contraceptives in both cohorts, those prescribed COCs from the IUD cohort, and those with concurrent IUDs from the COC cohort (using codes from Supplemental File 1, Table 1).

#### 2.2.2. Variables

We used propensity score matching to balance confounding variables between cohorts which have a known association with CeAD to minimize bias [28], considering variables within one year preceding and including the index date (Supplemental File 1, Table 3). To address potential temporal bias from differing years of cohort entry, we matched patients by their age at the index date and their current age (at the time of our query). This approach aimed to control for confounding related to the potentially increasing incidence of CeAD over time [2] and mitigate selection bias related to temporal trends in COCs versus IUDs [18].

#### 2.2.3. Primary outcome

We ascertained diagnoses of CeAD (ICD-10: I77.71 [carotid artery dissection] and I77.74 [vertebral artery dissection]) over a one-year follow-up window starting after the index date of inclusion. We used a one-year outcome considering contraceptive methods are typically used for months to years, and an increased risk of vascular adverse events has been shown to occur within the first year of COC use [24,29]. A shorter follow-up window was avoided as it may have identified insufficient events. A longer follow-up window would reduce available sample size and increase the potential for confounding, for example, related to possible discontinuation or use of different hormonal methods of contraception.

#### 2.2.4. Secondary outcomes

Secondarily, we examined time-to-event data by plotting cumulative incidence of CeAD per cohort with 95 % confidence intervals. Finally, we further evaluated the success of matching by calculating the RR for negative control outcomes unrelated to CeAD or COCs [30], targeting RR point estimates of 0.73 to 1.38, supportive of between-cohort balance [31]. These included evaluation and management codes (i.e., examination; CPT: 1013625) and administration of the human papillomavirus vaccine (CPT: 90650, 90,649, or 90,651). We also explored the post-matching RR for stroke incidence (ICD-10: I60, I61, I62, and I63). Each outcome was repeated for females prescribed at least two COCs (COC2).

#### 2.2.5. Statistical methods

We used the TriNetX platform software to explore the proportions, means, and standard deviations of baseline characteristics, and compared these variables using independent samples *t*-tests and Pearson χ-square tests. TriNetX calculated propensity scores via logistic regression using Python (scikit-learn version 1.3 [Python Software Foundation, Delaware, US]), with score of 1 representing the greatest likelihood of receiving an IUD. Patients were matched using a 1:1 ratio with greedy nearest-neighbor matching and caliper of 0.1 pooled standard deviations. We compared covariate balance using standardized mean difference (SMD) using a threshold of >0.2 for residual imbalance [32,33]. Length of record data were derived as a general descriptive measure and do not directly measure completely observable data following the index date. The RR for CeAD was calculated before and after matching by dividing the incidence of CeAD in the COC by the IUD cohort. We plotted propensity score density, incidence, and cumulative incidence of CeAD with 95 % confidence intervals using R (version 4.2.2, Vienna, AT [34]) and ggplot2 [35].

#### 2.2.6. Required study size

We calculated a total required size of 275,412 patients based on previous research [2,6] using G*Power (Kiel University, Germany) z-tests for examining a difference in proportion between cohorts of 0.0008 vs. 0.00021 to power our primary outcome using a power of 0.99, allocation ratio of one, and a two-tailed α error of 0.05.

## 3. Results

### 3.1. Patients

Before propensity matching there were 893,655 patients in the COC cohort and 214,766 in the IUD cohort. After matching, each cohort had 214,020 patients, with a mean age of approximately 31 years. Before matching, patients in the COC cohort had a younger mean age at the index date and query date, had lower incidence proportions of overweight or obese individuals, and individuals prescribed antimigraine agents (SMD > 0.2, Table 1). After matching, all variables were adequately balanced, including those which were reported for descriptive purposes only (SMD < 0.2).

### 3.2. Descriptive data

**Fig. 1.**
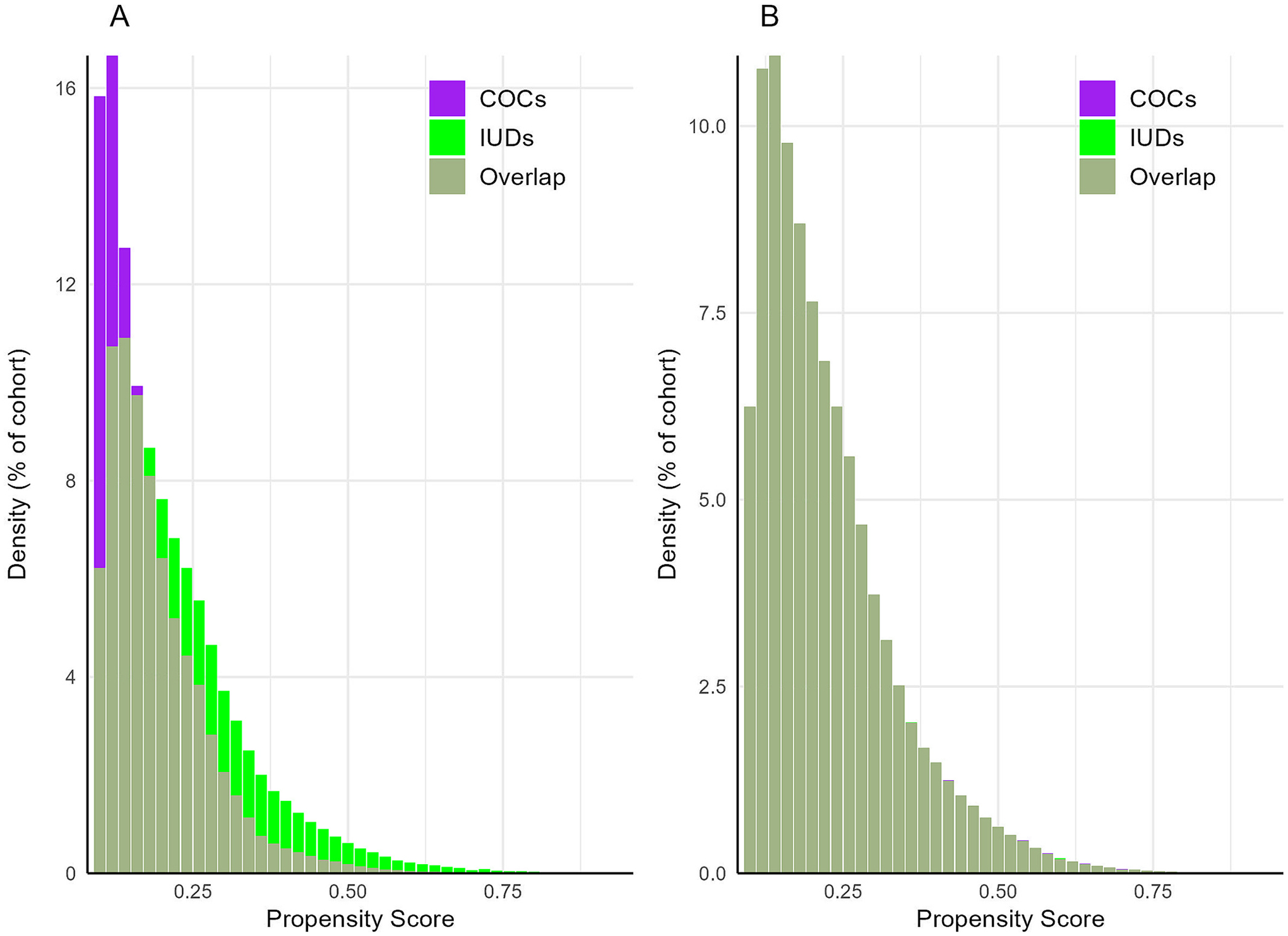
Propensity score density graph. Propensity scores before (A) and after (B) matching. The purple bars represent the combined oral contraceptives (COCs) cohort while the green bars represent the intrauterine devices (IUDs) cohort. Regions of propensity score density overlap, in which the shown proportion occurs in both cohorts, are shown by a darker shade of green. Following matching, propensity score densities overlap closely suggesting adequate covariate balance.

The mean number of facts (i.e., data points such as diagnoses, laboratory test results, and visits) per patient per cohort was adequate (COCs: 1108; IUDs: 1248). While there is no formal threshold and this measure varies contextually, studies using this data resource often consider facts values >1000 as sufficient [26,36,37]. A substantial proportion of each cohort had a length of record of at least 12 months (COCs: 95 %; IUD: 96 %). The proportion of patients with unknown age and unknown sex was 0 % in both cohorts. After matching, the plotted propensity score densities overlapped closely, indicating adequate covariate balance (Fig. 1). These results suggest there were negligible between-cohort differences with respect to data density, data completeness, and covariate balance.

### 3.3. Primary outcome

The incidence and risk of CeAD was greater among those prescribed COCs compared to matched controls with IUDs [95 % CI] (COCs: 0.016 %, IUDs: 0.008 %; RR 1.94 [1.10,3.43]; *P* = 0.0195). There were too few events to permit individual examination of incidence and RR for vertebral artery dissection and carotid dissection, considering the requirement to obfuscate small patient counts (i.e., *n* < 10) for de-identification purposes.

### 3.4. Secondary outcomes

Following matching, negative control outcomes were similar when comparing the COC to IUD cohort as defined by our a priori criteria, further suggesting successful balancing of cohorts. This included the incidence and RR for examination [95% confidence intervals] (53 % vs. 65 %; RR = 0.81 [0.81,0.82]) and human papillomavirus vaccine administration (0.9 % vs. 1.0 %; RR = 0.90 [0.84,0.95]). The proportion of patients who received an additional COC prescription during follow-up was 54 %, with a mean count of 2.6 prescriptions (standard deviation = 2.9). Including the COC prescribed on the index date, this equates to a mean of approximately 3.6 prescriptions per patient during the follow-up window.

Before matching, the incidence and risk of stroke was greater in the COC cohort compared to the IUD cohort, occurring in 663 patients with COCs versus 127 patients with IUDs (0.074 % vs. 0.059 %; RR = 1.26 [1.04,1.52]; *P* = 0.0189). After matching, the incidence and risk of stroke remained greater in the COC cohort compared to the IUD cohort, occurring in 227 patients with COCs versus 122 patients with IUDs (0.106 % vs. 0.057 %; RR = 1.86 [1.49,2.32]; *P* < 0.0001).

**Fig. 2.**
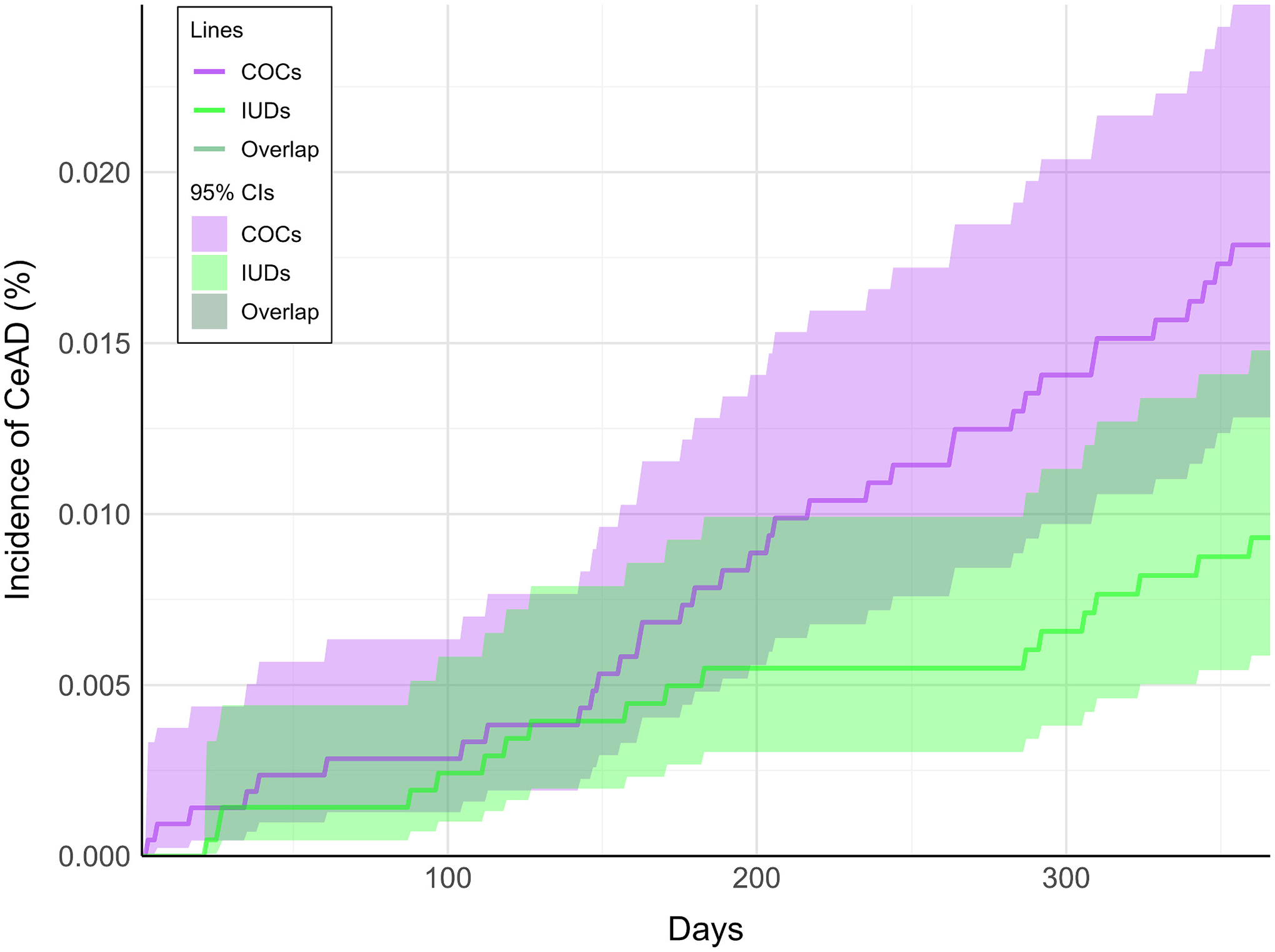
Cumulative incidence graph. Incidence curves for cervical artery dissection (CeAD) in the combined oral contraceptive cohort (COCs; purple lines) and intrauterine devices cohort (IUDs; green lines) are shown over the one-year follow-up period (365 days). Shaded ribbons indicate 95 % confidence intervals. Regions in which the lines or 95 % confidence intervals overlap between cohorts are shown in a darker shade of green.

**Fig. 3.**
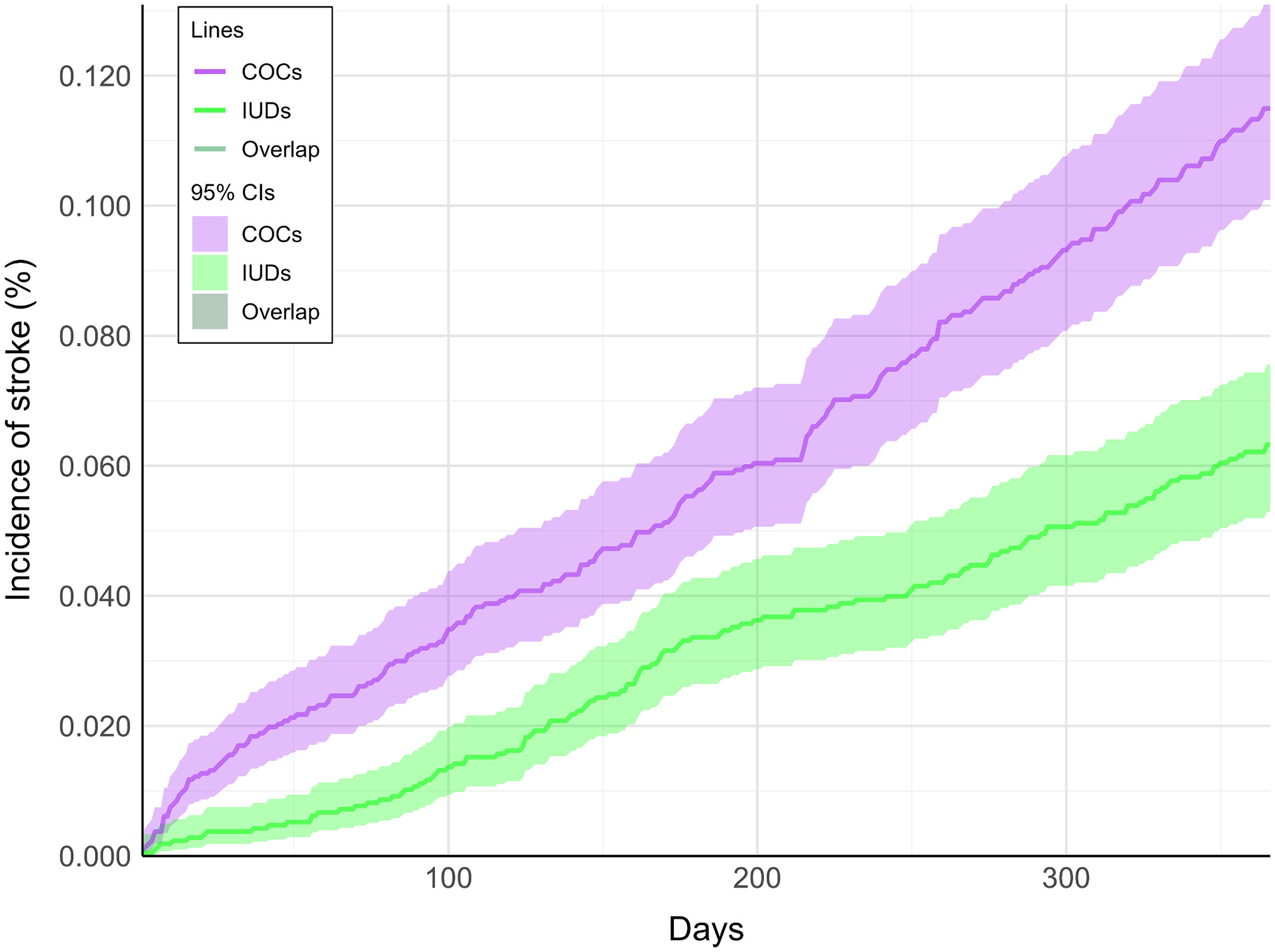
Cumulative incidence graph. Incidence curves for stroke in the combined oral contraceptive cohort (COCs; purple lines) and intrauterine devices cohort (IUDs; green lines) are shown over the one-year follow-up period (365 days). Shaded ribbons indicate 95 % confidence intervals. Regions in which the lines or 95 % confidence intervals overlap between cohorts are shown in a darker shade of green.

After matching, the cumulative incidence plots indicated a gradual increase in CeAD and stroke in the COC cohort compared to the IUD cohort (Fig. 2 and Fig. 3, respectively). While the 95 % confidence intervals for the cumulative incidence of CeAD overlapped at individual time points, our hypothesis and significance testing was based on the RR, which compares cumulative events over the entire follow-up period.

Similar to our main analysis, those prescribed at least two COCs (i.e., COC2 cohort) had an increase in risk of CeAD (RR = 1.83 [1.03,3.26]; *P* = 0.0357) and stroke (RR = 1.78 [1.41,2.25]; *P* < 0.0001) compared to the propensity-matched IUD cohort. We report outcomes and include cumulative incidence plots for this analysis in Supplemental File 2.

## 4. Discussion

The present findings support the hypothesis of a statistically significant increase in risk of CeAD among females prescribed COCs compared to matched controls using IUDs. Secondarily, we also identified a significantly increased risk of stroke associated with COCs. This secondary outcome corroborates our primary finding, CeAD being one cause of stroke, with 9 to 25 % of strokes generally being attributed to underlying CeAD in individuals under age 55 [38]. Although the magnitude of the RR point estimates for both CeAD and stroke were modest (i.e., 1.94 and 1.86, respectively), these conditions have potentially long-lasting neurological sequelae, suggesting our findings may represent clinically meaningful associations.

Our study’s findings of a positive association between COCs and CeAD risk align with one previous case-control study [7], but contrast with two others that found non-significant associations [8,9]. In addition, our results contrast those of Garg et al., who found no significant association between COCs and stroke attributed to CeAD [39]. However, our study builds on previous case-control studies which examined several risk factors in tandem in a broader population [7–9]. Our study represents the first cohort design to examine the COC-CeAD association, uses an active comparator design, controls for a broader range of confounding variables, focuses solely on females, and includes a larger sample size than previous studies. Despite these strengths, additional research is needed to corroborate our findings.

We observed a significant positive association between COC use and CeAD, with a similar association observed for stroke. The gradual increase in CeAD risk points to potential cumulative effects. While the underlying mechanisms remain unclear, several hypotheses warrant consideration. Given the increase in both risk of CeAD and stroke, prothrombotic effects of COCs may play a role [11]. COC-induced arterial collagen degeneration or chronic inflammation could contribute to vessel wall weakening over time, predisposing to CeAD [12,13]. A meta-analysis also identified a small, but statistically significant, time-dependent increase in risk of hypertension among those taking COCs [40], which could help explain our observed associations between both COCs and CeAD and stroke. Future research should explore these hypotheses, potentially through cross-sectional studies comparing blood pressure measurements and inflammatory markers in COC users and non-users who experience CeAD.

The incidence of CeAD in our study aligns with recent epidemiological data, though direct comparisons should be interpreted cautiously due to methodological differences. Our inclusion of adolescents, focus on large medical centers, exclusion of emergency care settings, and recent data range (2014–2024) variably affect our mean population age and observed CeAD and stroke incidences. In our unmatched IUD cohort, CeAD incidence (8.8 per 100,000 person-years) aligns with a US study which reported a CeAD incidence of 10.17 per 100,000 person-years from 2017 to 2020 among adult females [2]. This similarity suggests those using IUDs are not at increased risk of CeAD, supporting our choice of this active control and aligning previous research which suggested that IUDs did not increase the risk of adverse vascular events [6,15].

COCs are typically dispensed for 1 or 3 months, with extended durations up to 6 or 12 months being less common [41,42]. Therefore, our estimate of 3.6 COC prescriptions per patient may correspond to as few as 3.6 to 10.8 months of COC use following the index date. Due to variability in prescription durations and the lack of specific data on the exact durations, this estimate should be interpreted cautiously. Our real-world study, analogous to an “intent to treat” strategy, aimed to minimize biases related to requiring multiple COC prescriptions. Chiefly, requiring more than one prescription could inadvertently exclude patients who developed CeAD after their first COC prescription. Despite not requiring continued use or compliance, we identified a significant COC-CeAD association. Finally, our findings were corroborated by similar results in a secondary analysis of females prescribed at least two COCs. Future studies should explore a dose-response relationship between COC duration and CeAD, potentially using a case-control design.

At baseline, patients in the IUD cohort were older and generally had more comorbidities compared to those in the COC cohort, indicating a higher baseline risk of CeAD. Before matching, the incidence of CeAD was only modestly higher in the COC cohort compared to the IUD cohort, and the RR did not reach statistical significance. After matching, which paired younger and healthier IUD patients with COC users, the magnitude of between-cohort difference in CeAD incidence became more pronounced and the RR reached statistical significance. This change highlights the value of propensity score matching in mitigating confounding due to healthy user bias among COC users. By creating a more comparable control group with similar baseline characteristics, our methods improved the validity of the comparison, as further supported by low SMDs across covariates after matching [21,22].

The observed association between COCs and CeAD identified in this study should be viewed as preliminary and cannot replace the complexity of shared decision-making and clinical judgement regarding contraceptive use. Both COCs and IUDs have relevant advantages, contraindications, and potential risks which are beyond the scope of CeAD discussed in this study [43]. In addition to the need to corroborate our findings, the larger weight of evidence as well as patient preferences should be prioritized when making medical decisions regarding contraceptive use.

Given our identification of an independent positive association between COCs and CeAD, future studies could explore interactions between COCs and factors like smoking, obesity, or age. Regression modeling in a case-control design may better enable examination of subgroup-specific risks or mediating factors compared to the matched cohort approach used herein. Such efforts could clarify whether the observed association varies by age or comorbidity subgroups.

### 4.1. Strengths and limitations

Strengths of this study include a large sample size, matching strategy and new-user design features, registered protocol, and interdisciplinary investigator team. However, several limitations are present. We are unable to manually review charts to corroborate the data query. There may be unmeasured confounding related to genetic factors and dose and/or type of COC/IUD used. While the positive predictive value of ICD codes for CeAD has been reported to be 90 % [44], the overall accuracy of our outcome remains unclear. Regardless, there is no reason to suspect bias due to differential outcome documentation between cohorts. The present study findings may only generalize to females upon initial COC prescription rather than those prescribed COCs for greater than one year, and do not apply to other forms of hormonal contraception. Requirements for a preceding and follow-up visit used a general, flexible timing, which may have varied between cohorts.

In general, the risk of vascular adverse events may increase with a longer duration of COCs [24]. Accordingly, future research could consider examining the association between COC duration and CeAD likelihood, as well as the association between CeAD and other forms of hormonal birth control.

Our observed effect estimate for stroke should be interpreted with some caution, as our propensity matching and selection criteria were primarily tailored to detect differences in CeAD and these conditions have different risk factors [3]. Furthermore, we were unable to distinguish between different subtypes of stroke. Given the larger number of strokes relative to CeAD in the matched COC cohort, CeAD would only account for at most 5.3 % of strokes, with the remainder having other etiologies. These remain beyond the scope and capacity of our study to examine in detail.

A prior meta-analysis examining the association between COCs and stroke found that stronger doses of estrogen yielded larger magnitude of association with stroke risk relative to lower estrogen doses [20]. Accordingly, there may be varying degrees of CeAD risk dependent on the type of COC composition or its dose. Further studies may seek to determine whether there is a dose-response association between various COC estrogen doses and CeAD.

## 5. Conclusions

Our results support the hypothesis of a positive association between COCs and CeAD and corroborate the existing literature regarding the positive COC-stroke association. However, as the current literature regarding COCs and CeAD remains limited, additional research is needed to verify our preliminary observations as well as explore longer follow-up windows, examine possible pathophysiological mechanisms promoting CeAD among those using COCs, and test possible dose-response relationships between COC duration or estrogen dose and CeAD incidence. These findings do not supersede the broader context of contraceptive decision-making, and patients and clinicians should consider the overall efficacy, risks, and preferences of various contraceptive methods.

## Supplementary Material

1

2

## Figures and Tables

**Table 1:** Baseline characteristics before and after matching. This table shows variables found in over 5 % of both cohorts after matching. Additional variables are shown in the Supplemental File Table 3.

Variable	Before matching			After matching		
Variable (n (%) or mean (SD))	COCs	IUDs	SMD	COCs	IUDs	SMD
N	893,655	214,766	NA	214,020	214,020	NA
Age at Index Date	27.5 (9.1)	31.2 (8.5)	0.413	31.3 (8.6)	31.1 (8.5)	0.013
Age at Query Date	33.4 (9.6)	36.6 (8.9)	0.355	36.7 (9.0)	36.6 (8.8)	0.014
Hypertensive Diseases	23,175 (3 %)	13,329 (6 %)	0.177	13,029 (6 %)	12,977 (6 %)	0.001
Acute Upper Respiratory Infections	84,246 (9 %)	20,349 (9 %)	0.002	19,969 (9 %)	20,236 (9 %)	0.004
Overweight And Obesity	55,841 (6 %)	27,826 (13 %)	0.229	26,792 (13 %)	27,305 (13 %)	0.007
Not Hispanic or Latino	603,174 (67 %)	146,843 (68 %)	0.019	145,478 (68 %)	146,240 (68 %)	0.008
Antimigraine Agents	38,691 (4 %)	23,241 (11 %)	0.247	23,358 (11 %)	22,794 (11 %)	0.008
Migraine	34,143 (4 %)	13,969 (7 %)	0.122	14,829 (7 %)	13,743 (6 %)	0.020
Unknown Race	88,148 (10 %)	23,073 (11 %)	0.029	21,114 (10 %)	23,040 (11 %)	0.030
White	621,755 (70 %)	142,707 (66 %)	0.067	146,354 (68 %)	142,224 (66 %)	0.041
Black or African American	94,123 (11 %)	29,920 (14 %)	0.104	24,115 (11 %)	29,728 (14 %)	0.079
Unknown Ethnicity	200,345 (22 %)	38,725 (18 %)	0.109	46,115 (22 %)	38,637 (18 %)	0.088
Hispanic or Latino	90,136 (10 %)	29,198 (14 %)	0.109	22,427 (10 %)	29,143 (14 %)	0.097

Aside from data regarding age, and number of patients (N), the remaining rows are sorted by ascending post-matching standardized mean difference (SMD). Race and ethnicity were not matched and are reported solely for descriptive purposes. Other abbreviations: combined oral contraceptives (COCs), intrauterine devices (IUDs), not applicable (NA), standard deviation (SD).

**Table 2:** Incidence and risk ratio of cervical artery dissection.

	Before matching		After matching^a^	
	COCs	IUDs	COCs	IUDs
Number of patients	893,655	214,766	214,020	214,020
CeAD n (%)	94 (0.011 %)	19 (0.009 %)	35 (0.016 %)	18 (0.008 %)
CeAD n per 100,000 person-years	10.5	8.8	16.4	8.4
CeAD risk ratio (95 % CI)	1.19 (0.73,1.95); *P* = 0.4908	Reference	1.94 (1.10,3.43); *P* = 0.0195	Reference

Abbreviations: Cervical artery dissection (CeAD), combined oral contraceptives (COCs), intrauterine devices (IUDs).

a Data used for primary outcome.

## Data Availability

Minimal, de-identified, aggregate datasets used to report primary outcomes and plot cumulative incidence and propensity score density are available in a Figshare repository (https://doi.org/10.6084/m9.figshare.26517367 [45]).

## Appendix A. Supplementary data

Supplementary data to this article can be found online at https://doi.org/10.1016/j.thromres.2025.109279.
