# Supplementary material for: Association between combined oral contraceptive prescription and cervical artery dissection: A retrospective cohort study
Source: Thromb Res. Author manuscript; Available in PMC 2026 May 12. (PMC13163211; doi:10.1016/j.thromres.2025.109279)
Supplement: 1 [file NIHMS2171222-supplement-1.pdf]

Supplemental File 1

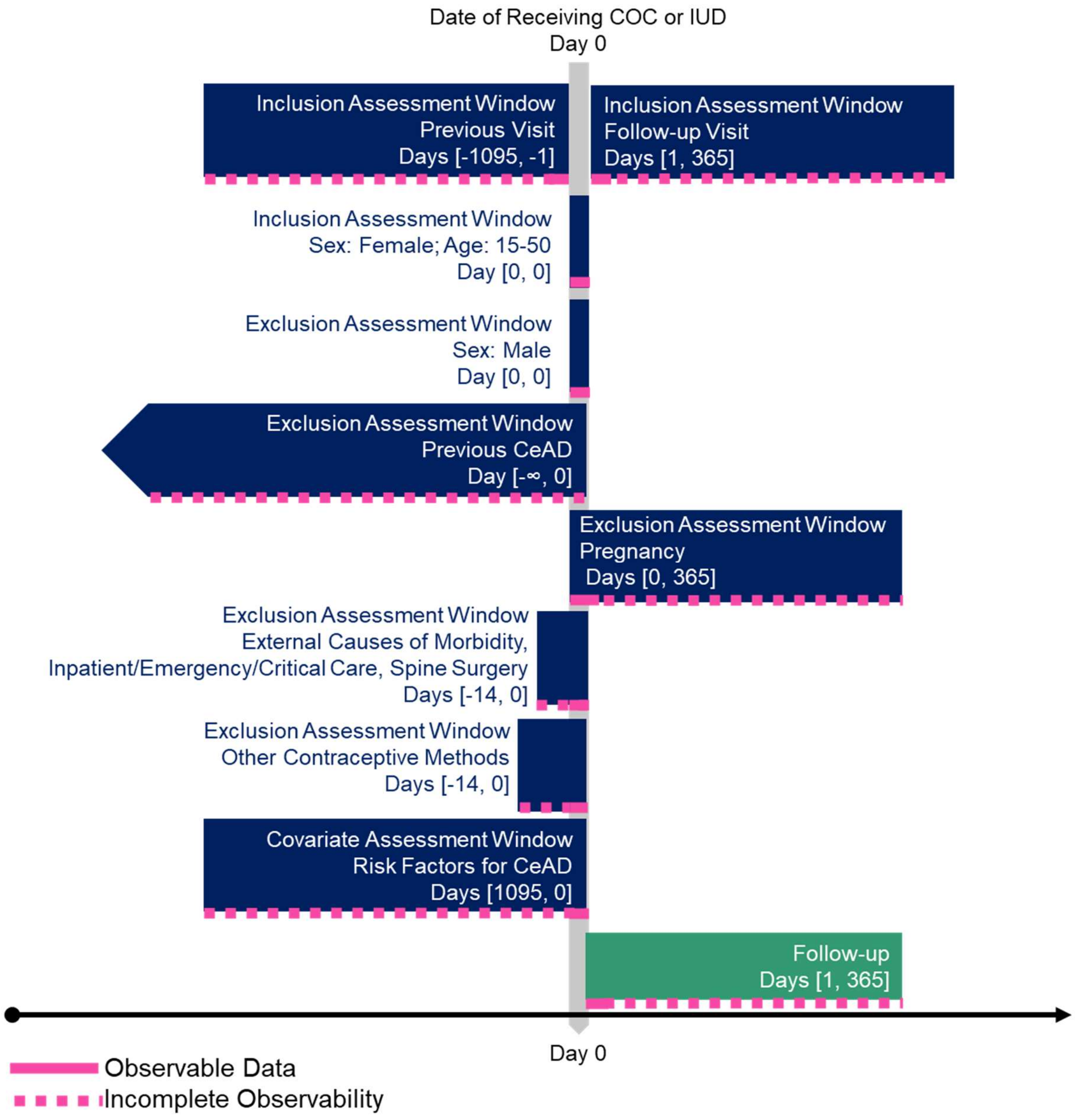

Figure 1: Graphical depiction of study design. The vertical grey arrow represents the index date (cohort entry date, day 0), defined as the start of combined oral contraceptive prescriptions (COCs) or intrauterine devices (IUDs). Text and boxes indicate study selection criteria which were assessed during time windows ([#, #]) of days in relation to the index date. The washout period for previous cervical artery dissection (CeAD) spanned as long as preceding data were available per patient (-∞). Image created using Creative Commons template from Wang et al.[1]

Table 1: Codes used for inclusion criteria

Codes	Definition and filters
Combined oral contraceptive (RxNorm)	
4124	Ethinyl estradiol
7514, 6373, 31994, 11636, 22656, 7518	Progestins: Norethindrone, levonorgestrel, norgestimate, drospirenone, desogestrel, norgestrel
Intrauterine device	
Z30.430 (ICD-10)	Encounter for insertion of intrauterine contraceptive device
58300 (CPT)	Insertion of intrauterine device
0UH97HZ (ICD-10-PCS)	Insertion of contraceptive device into uterus, via natural or artificial opening
J7300 (HCPCS)	Intrauterine contraceptive device
J7301 (HCPCS)	Levonorgestrel-releasing intrauterine contraceptive system (Skyla), 13.5 mg
J7302 (HCPCS)	Levonorgestrel-releasing intrauterine contraceptive system, 52 mg (depreciated 2015)
J7296 (HCPCS)	Levonorgestrel-releasing intrauterine contraceptive system (Kyleena®) 19.5 mg
J7297 (HCPCS)	Levonorgestrel-releasing intrauterine contraceptive system (Liletta®) 52 mg
J7298 (HCPCS)	Levonorgestrel-releasing intrauterine contraceptive system (Mirena®) 52 mg
Q9984 (HCPCS)	Levonorgestrel-releasing intrauterine contraceptive system (Kyleena®) 19.5 mg (depreciated 2017)
65200003 (SNOMED)	Insertion of intrauterine contraceptive device
472837007 (SNOMED)	Insertion of hormone-releasing intrauterine contraceptive device
6373 (RxNorm)	Levonorgestrel; using filters: Kyleena, Liletta, Mirena, or Skyla
Abbreviations: Current Procedural Terminology (CPT), Healthcare Common Procedure Coding System (HCPCS), normalized drug names (RxNorm), Systematized Nomenclature Of Medicine (SNOMED), Veterans Affairs National Drug File (VA), International Classification of Diseases, Tenth Revision (ICD-10)	

Table 2: Exclusion criteria for both cohorts

Code(s)	Definition	Window (days)
Diagnoses		
I77.71 (ICD-10)	Dissection of carotid artery	-∞ to 0
I77.74 (ICD-10)	Dissection of vertebral artery	-∞ to 0
I77.75 (ICD-10)	Dissection of other precerebral arteries	-∞ to 0
G45 (ICD-10)	Transient cerebral ischemic attacks and related syndromes	-∞ to 0
G46 (ICD-10)	Vascular diseases of brain in cerebrovascular diseases	-∞ to 0
I60-I69 (ICD-10)	Cerebrovascular diseases	-∞ to 0
I97.81 (ICD-10)	Intraoperative cerebral infarction	-∞ to 0
I97.82 (ICD-10)	Postoperative cerebral infarction	-∞ to 0
S15.0 (ICD-10)	Injury of carotid artery of neck	-∞ to 0
S15.0 (ICD-10)	Injury of vertebral artery	-∞ to 0
I69 (ICD-10)	Sequelae of cerebrovascular diseases (i.e., late effects)	-∞ to 0
Z86.73 (ICD-10)	Personal history of transient ischemic attack (TIA), and cerebral infarction without residual deficits	-∞ to 0
V00-Y99 (ICD-10)	External causes of morbidity	-14 to 0
O00-O9A (ICD-10)	Pregnancy, childbirth, and the puerperium	0 to 365
Procedures and visits		
1022277 (CPT)	Transcatheter placement of extracranial vertebral artery stent(s), including radiologic supervision and interpretation, open or percutaneous	-∞ to 0
1022228 (CPT)	Transcatheter placement of intravascular stent(s), cervical carotid artery, open or percutaneous, including angioplasty, when performed, and radiological supervision and interpretation	-∞ to 0
35301 (CPT)	Thrombarterectomy, including patch graft, if performed; carotid, vertebral, subclavian, by neck incision	-∞ to 0
03QJ, 03QH, 03QN, 03QM, 03QL, 03QK (ICD-10-PCS)	Carotid artery repair	-∞ to 0
03QQ, 03QP (ICD-10-PCS)	Vertebral artery repair	-∞ to 0
1013711 (CPT)	Emergency department services	-14 to 0
1013699 (CPT)	Inpatient or observation consultations	-14 to 0
1013729 (CPT)	Critical care services	-14 to 0
1004038 (CPT)	Surgical procedures on the spine (vertebral column)	-14 to 0
1009340 (CPT)	Surgical procedures on the spine and spinal cord	-14 to 0
Other contraceptive methods		
HS200 (VA)	Contraceptives: Filters: vaginal product, injectable product, or drug implant product (also excluded oral product in intrauterine device cohort)	-90 to 0
G03A (ATC)	Hormonal contraceptives: Filters: drug implant product, injectable product, topical product, or vaginal product (also excluded oral product in intrauterine device cohort)	-90 to 0
Abbreviations: any preceding duration of time available in the patient's data (-∞), Anatomical Therapeutic Chemical (ATC) Classification, Current Procedural Terminology (CPT); International Classification of Diseases, 10th Revision (ICD-10); ICD-10 Procedure Coding System (ICD-10-PCS), Veterans Affairs National Drug File (VA)		

Table 3: Variables controlled for in propensity score matching

Variable	Description	Association with CeAD	Reference(s)
Age	Age at index and current age	NA	
Diagnoses (ICD-10)			
J00-J06	Acute upper respiratory infections	⬆	[2,3]
E88.01	Alpha-1-antitrypsin deficiency	⬆	[4]
Z55-Z65	Adverse socioeconomic and psychosocial circumstances	⬇	[2,5]
I71	Aortic aneurysm and dissection	⬆	[6]
I77.3	Arterial fibromuscular dysplasia	⬆	[7]
E06.3	Autoimmune thyroiditis	⬆	[8,9]
E08-E13	Diabetes mellitus	⬇	[10–12]
I70-I79	Diseases of arteries, arterioles, and capillaries (includes aneurysms, arterial embolism, arteritis, and arterial fibromuscular dysplasia)	⬆	[6,7]
Q79.6	Ehlers-Danlos syndromes	⬆	[7]
R79.82	Elevated C-reactive protein (CRP)	⬆	[13]
R70	Elevated erythrocyte sedimentation rate and abnormality of plasma viscosity	⬆	[13]
D72.82	Elevated white blood cell count	⬆	[14]
Z82.4	Family history of ischemic heart disease and other diseases of the circulatory system	⬆	[10]
E72.11	Homocystinuria	⬆	[15]
E78.5	Hyperlipidemia, unspecified	⬇	[10,11,16]
I10-I1A	Hypertensive diseases	⬆	[10]
F10-F19	Mental and behavioral disorders due to psychoactive substance use	⬆	[17,18]
G43	Migraine	⬆	[10]
Q78.0	Osteogenesis imperfecta	⬆	[7]
D68	Other coagulation defects (includes antiphospholipid syndrome, lupus anticoagulant syndrome)	⬆	[8,19]
Q87	Other specified congenital syndromes (includes Marfan syndrome, Loeys Dietz syndrome, Alport syndrome, arterial tortuosity syndrome)	⬆	[7,20]
E66	Overweight and obesity	⬇	[10]
M30-M36	Systemic connective tissue disorders (includes lupus, Sjögren's syndrome, hypermobility syndrome)	⬆	[21]
Z72.0	Tobacco use	⬆	[10,11]
Procedures/treatments			
CV490 (VA)	Antihypertensives, other	⬇	[10,16]
CN105 (VA)	Antimigraine agents (includes triptans)	⬆	[22]
CV100 (VA)	Beta blockers/related	⬇	[10]
AM400 (VA)	Quinolones (includes fluoroquinolones)	⬆	[23]
Abbreviations: Cervical artery dissection (CeAD), International Classification of Diseases, 10th Edition (ICD-10), Veterans Affairs National Drug File (VA), positive association (⬆), negative association (⬇)			

Table 4: Baseline characteristics before and after matching.

Variable	Before matching			After matching		
Variable (n (%) or mean (SD))	COCs	IUDs	SMD	COCs	IUDs	SMD
N	893,655	214,766	NA	214,020	214,020	NA
Age at Index Date	27.5 (9.1)	31.2 (8.5)	0.413	31.3 (8.6)	31.1 (8.5)	0.013
Age at Query Date	33.4 (9.6)	36.6 (8.9)	0.355	36.7 (9.0)	36.6 (8.8)	0.014
Osteogenesis Imperfecta	70 (0%)	13 (0%)	0.002	14 (0%)	13 (0%)	0.001

Variable	Before matching			After matching		
Variable (n (%) or mean (SD))	COCs	IUDs	SMD	COCs	IUDs	SMD
Alpha-1-Antitrypsin Deficiency	45 (0%)	12 (0%)	0.001	13 (0%)	12 (0%)	0.001
Hypertensive Diseases	23175 (3%)	13329 (6%)	0.177	13029 (6%)	12977 (6%)	0.001
Substance Use Disorder	24623 (3%)	10293 (5%)	0.107	10126 (5%)	10079 (5%)	0.001
Ehlers-Danlos Syndromes	1255 (0%)	317 (0%)	0.002	320 (0%)	311 (0%)	0.001
Native Hawaiian / Pacific Islander	1952 (0%)	530 (0%)	0.006	517 (0%)	529 (0%)	0.001
Elevated C-Reactive Protein	587 (0%)	190 (0%)	0.008	179 (0%)	187 (0%)	0.001
Diabetes Mellitus	13096 (1%)	5732 (3%)	0.085	5550 (3%)	5627 (3%)	0.002
Tobacco Use	3688 (0%)	2114 (1%)	0.069	1982 (1%)	2030 (1%)	0.002
Homocystinuria	99 (0%)	44 (0%)	0.007	34 (0%)	42 (0%)	0.003
Elevated White Blood Cell Count	4148 (0%)	1434 (1%)	0.027	1359 (1%)	1409 (1%)	0.003
American Indian or Alaska Native	2882 (0%)	743 (0%)	0.004	696 (0%)	743 (0%)	0.004
Adverse Socioeconomic & Psychosocial Factors	9423 (1%)	4742 (2%)	0.091	4486 (2%)	4613 (2%)	0.004
Acute Upper Respiratory Infections	84246 (9%)	20349 (9%)	0.002	19969 (9%)	20236 (9%)	0.004
Autoimmune Thyroiditis	5779 (1%)	1461 (1%)	0.004	1367 (1%)	1450 (1%)	0.005
Hyperlipidemia, Unspecified	11467 (1%)	4428 (2%)	0.061	4213 (2%)	4357 (2%)	0.005
Arterial Fibromuscular Dysplasia	36 (0%)	20 (0%)	0.006	10 (0%)	19 (0%)	0.005
Elevated Erythrocyte Sedimentation Rate	389 (0%)	136 (0%)	0.009	104 (0%)	132 (0%)	0.006
Quinolones	31969 (4%)	7693 (4%)	0.000	7427 (3%)	7649 (4%)	0.006
Aortic Aneurysm and Dissection	155 (0%)	65 (0%)	0.008	41 (0%)	62 (0%)	0.006
Overweight And Obesity	55841 (6%)	27826 (13%)	0.229	26792 (13%)	27305 (13%)	0.007
Not Hispanic or Latino	603174 (67%)	146843 (68%)	0.019	145478 (68%)	146240 (68%)	0.008
Diseases Of Arteries, Arterioles and Capillaries	4964 (1%)	1628 (1%)	0.025	1450 (1%)	1595 (1%)	0.008
Antimigraine Agents	38691 (4%)	23241 (11%)	0.247	23358 (11%)	22794 (11%)	0.008
Systemic Connective Tissue Disorders	4007 (0%)	1858 (1%)	0.052	1614 (1%)	1784 (1%)	0.009
Family History of Ischemic Heart Disease	5571 (1%)	2994 (1%)	0.077	2595 (1%)	2866 (1%)	0.011
Other Specified Congenital Malformation Syndromes	616 (0%)	213 (0%)	0.010	133 (0%)	212 (0%)	0.013
Migraine	34143 (4%)	13969 (7%)	0.122	14829 (7%)	13743 (6%)	0.020
Other Coagulation Defects	1542 (0%)	2013 (1%)	0.103	1316 (1%)	1700 (1%)	0.021
Beta Blockers/Related	27036 (3%)	11560 (5%)	0.118	10250 (5%)	11276 (5%)	0.022
Asian	36547 (4%)	8327 (4%)	0.011	9343 (4%)	8316 (4%)	0.024
Antihypertensives	9727 (1%)	5260 (2%)	0.103	4272 (2%)	5064 (2%)	0.025
Unknown Race	88148 (10%)	23073 (11%)	0.029	21114 (10%)	23040 (11%)	0.030
White	621755 (70%)	142707 (66%)	0.067	146354 (68%)	142224 (66%)	0.041
Other Race	48248 (5%)	9466 (4%)	0.046	11881 (6%)	9440 (4%)	0.052
Black or African American	94123 (11%)	29920 (14%)	0.104	24115 (11%)	29728 (14%)	0.079
Unknown Ethnicity	200345 (22%)	38725 (18%)	0.109	46115 (22%)	38637 (18%)	0.088
Hispanic or Latino	90136 (10%)	29198 (14%)	0.109	22427 (10%)	29143 (14%)	0.097

Aside from data regarding age, and number of patients (N), the remaining rows are sorted by ascending post-matching standardized mean difference (SMD). Race and ethnicity were not matched and are reported solely for descriptive purposes. Other abbreviations: combined oral contraceptives (COCs), intrauterine devices (IUDs), not applicable (NA), standard deviation (SD).

References

1. Wang SV, Schneeweiss S: A Framework for Visualizing Study Designs and Data Observability in Electronic Health Record Data. Clin Epidemiol. 2022, 14:601–8. 10.2147/CLEP.S358583

2. Grau AJ, Brandt T, Buggle F, et al.: Association of Cervical Artery Dissection With Recent Infection. Arch Neurol. 1999, 56:851–6. 10.1001/archneur.56.7.851

3. Witsch J, Rutrick SB, Lansdale KN, et al.: Influenza-Like Illness as a Short-Term Risk Factor for Arterial Dissection. Stroke. 2023, 54:e66–8. 10.1161/STROKEAHA.122.042367

4. Pezzini A, Magoni M, Corda L, et al.: Alpha-1-antitrypsin deficiency-associated cervical artery dissection: report of three cases. Eur Neurol. 2002, 47:201–4. 10.1159/000057899

5. Kellert L, Grau A, Pezzini A, et al.: University education and cervical artery dissection. J Neurol. 2018, 265:1065–70. 10.1007/s00415-018-8798-7

6. Witsch J, Mir SA, Parikh NS, et al.: Association Between Cervical Artery Dissection and Aortic Dissection. Circulation. 2021, 144:840–2. 10.1161/CIRCULATIONAHA.121.055274

7. Debette S: Pathophysiology and risk factors of cervical artery dissection: what have we learnt from large hospital-based cohorts? Curr Opin Neurol. 2014, 27:20–8. 10.1097/WCO.0000000000000056

8. Li H, Song P, Yang W, et al.: Association Between Autoimmune Diseases and Spontaneous Cervicocranial Arterial Dissection. Front Immunol. 2022, 12:. 10.3389/fimmu.2021.820039

9. Pezzini A, Del Zotto E, Mazziotti G, et al.: Thyroid Autoimmunity and Spontaneous Cervical Artery Dissection. Stroke. 2006, 37:2375–7. 10.1161/01.STR.0000236500.15976.f3

10. Del Zotto E, Grassi M, Zedde M, et al.: Risk profile of patients with spontaneous cervical artery dissection. Ann Neurol. 2023, 94:585–95.

11. Abdelnour LH, Abdalla ME, Elhassan S, Kheirelseid EAH: Meta-analysis of cardiovascular risk profile of stroke secondary to spontaneous cervical artery dissection compared to ischemic stroke of other causes. Health Sci Rev. 2022, 5:100058. 10.1016/j.hsr.2022.100058

12. Engelter ST, Grond-Ginsbach C, Metso TM, et al.: Cervical artery dissection: trauma and other potential mechanical trigger events. Neurology. 2013, 80:1950–7. 10.1212/WNL.0b013e318293e2eb

13. Naggara O, Touzé E, Marsico R, et al.: High-resolution MR imaging of periarterial edema associated with biological inflammation in spontaneous carotid dissection. Eur Radiol. 2009, 19:2255–60. 10.1007/s00330-009-1415-5

14. Grond-Ginsbach C, Giossi A, Aksay SS, et al.: Elevated peripheral leukocyte counts in acute cervical artery dissection. Eur J Neurol. 2013, 20:1405–10. 10.1111/ene.12201

15. Gallai V, Caso V, Paciaroni M, Cardaioli G, Arning E, Bottiglieri T, Parnetti L: Mild Hyperhomocyst(e)inemia. Stroke. 2001, 32:714–8. 10.1161/01.STR.32.3.714

16. Abdelnour LH, Abdalla ME, Elhassan S, Kheirelseid EAH: Diabetes, hypertension, smoking, and hyperlipidemia as risk factors for spontaneous cervical artery dissection: Meta-analysis of case-control studies. Curr J Neurol. 2022, 21:183–93. 10.18502/cjn.v21i3.11112

17. Hori S, Hori E, Umemura K, et al.: Anatomical Variations of Vertebrobasilar Artery are Closely Related to the Occurrence of Vertebral Artery Dissection—An MR Angiography Study. J Stroke Cerebrovasc Dis. 2020, 29:104636. 10.1016/j.jstrokecerebrovasdis.2020.104636

18. Smith WS, Johnston SC, Skalabrin EJ, Weaver M, Azari P, Albers GW, Gress DR: Spinal manipulative therapy is an independent risk factor for vertebral artery dissection. Neurology. 2003, 60:1424–8. 10.1212/01.WNL.0000063305.61050.E6

19. Al-banaa K, Alshaikhli A, AL-Hareeri A, Abdelhalim M, Al-Hillan A, Joshi T: Arterial Dissection in Antiphospholipid Syndrome Patients: Two Case Reports and a Literature Review. Eur J Case Rep Intern Med. 2021, 8:002610. 10.12890/2021_002610

20. Traenka C, Kloss M, Strom T, et al.: Rare genetic variants in patients with cervical artery dissection. Eur Stroke J. 2019, 4:355–62. 10.1177/2396987319861869

21. Li H, Xu S, Xu B, Zhang Y, Yin J, Yang Y: Unraveling the Links between Chronic Inflammation, Autoimmunity, and Spontaneous Cervicocranial Arterial Dissection. J Clin Med. 2023, 12:5132. 10.3390/jcm12155132

22. Roberto G, Piccinni C, D'Alessandro R, Poluzzi E: Triptans and serious adverse vascular events: Data mining of the FDA Adverse Event Reporting System database. Cephalalgia. 2014, 34:5–13. 10.1177/0333102413499649

23. Zotto ED, Pezzini A: Use of fluoroquinolones and the risk of spontaneous cervical artery dissection. Eur J Neurol. 2019, 26:1028–31. https://doi.org/10.1111/ene.13917
