# Supplementary material for: Association between combined oral contraceptive prescription and cervical artery dissection: A retrospective cohort study
Source: Thromb Res. Author manuscript; Available in PMC 2026 May 12. (PMC13163211; doi:10.1016/j.thromres.2025.109279)
Supplement: 2 [file NIHMS2171222-supplement-2.pdf]

Supplemental File 2

Combined oral contraceptives (≥2 prescriptions: COC2) compared to intrauterine devices

This sensitivity analysis explores a similar comparison to our main analysis, with the only change being the combined oral contraceptive (COC) cohort was required to have at least two prescriptions of COCs (i.e., COC2). The other methods including the data source, query date (October 16, 2024), data range, selection criteria, and propensity matched variables remained the same as our main analysis. The intrauterine devices (IUD) cohort was the same before matching in terms of patient count, however, there were some differences in patient counts following matching. Overall, the results of this analysis yielded similar results to our main analysis.

Descriptive data

- COC2 cohort size
    - Before matching: 457,386
    - After matching: 201,372
- IUDs cohort size
    - Before matching: 214,766
    - After matching: 201,372
- Data quality for COC2 cohort:
    - Data density: 1,095 facts per patient
    - Length of record: 96% of patients have data spanning at least 12 months

Table 1: Baseline characteristics before and after matching

Variable	Before matching			After matching		
Variable (n (%) or mean (SD))	COC2	IUDs	SMD	COC2	IUDs	SMD
N	457,386	214,766	NA	201,372	201,372	NA
Age At Index Date	26.9 (9.0)	31.2 (8.5)	0.485	31.2 (8.9)	30.7 (8.4)	0.048
Age at Query Date	32.9 (9.6)	36.6 (8.9)	0.407	36.7 (9.3)	36.3 (8.8)	0.046
Autoimmune Thyroiditis	2656 (1%)	1461 (1%)	0.013	1301 (1%)	1318 (1%)	0.001
Arterial Fibromuscular Dysplasia	18 (0%)	20 (0%)	0.007	16 (0%)	14 (0%)	0.001
Osteogenesis Imperfecta	34 (0%)	13 (0%)	0.002	15 (0%)	13 (0%)	0.001
Alpha-1-Antitrypsin Deficiency	21 (0%)	12 (0%)	0.001	12 (0%)	10 (0%)	0.001
Ehlers-Danlos Syndromes	573 (0%)	317 (0%)	0.006	268 (0%)	280 (0%)	0.002
Elevated White Blood Cell Count	1920 (0%)	1434 (1%)	0.034	1155 (1%)	1181 (1%)	0.002
Elevated C-Reactive Protein	238 (0%)	190 (0%)	0.014	147 (0%)	157 (0%)	0.002
Acute Upper Respiratory Infections	42296 (9%)	20349 (9%)	0.008	18334 (9%)	18462 (9%)	0.002
Homocystinuria	47 (0%)	44 (0%)	0.008	29 (0%)	36 (0%)	0.003
Substance Use Disorder	9958 (2%)	10293 (5%)	0.143	7817 (4%)	7928 (4%)	0.003
Aortic Aneurysm and Dissection	52 (0%)	65 (0%)	0.013	37 (0%)	50 (0%)	0.004
Diseases Of Arteries, Arterioles and Capillaries	2170 (0%)	1628 (1%)	0.036	1260 (1%)	1349 (1%)	0.006
Systemic Connective Tissue Disorders	1704 (0%)	1858 (1%)	0.063	1321 (1%)	1414 (1%)	0.006
Migraine	15524 (3%)	13969 (7%)	0.144	11564 (6%)	11298 (6%)	0.006
Quinolones	16443 (4%)	7693 (4%)	0.001	7256 (4%)	7038 (3%)	0.006
Hyperlipidemia, Unspecified	5089 (1%)	4428 (2%)	0.076	3307 (2%)	3479 (2%)	0.007
Native Hawaiian / Pacific Islander	903 (0%)	530 (0%)	0.010	424 (0%)	490 (0%)	0.007
Tobacco Use	1393 (0%)	2114 (1%)	0.085	1272 (1%)	1407 (1%)	0.008
American Indian or Alaska Native	1352 (0%)	743 (0%)	0.009	599 (0%)	698 (0%)	0.009
Other Specified Congenital Malformation Syndromes	287 (0%)	213 (0%)	0.013	140 (0%)	191 (0%)	0.009
Elevated Erythrocyte Sedimentation Rate	152 (0%)	136 (0%)	0.014	73 (0%)	113 (0%)	0.009
Unknown Ethnicity	86119 (19%)	38725 (18%)	0.021	36080 (18%)	36800 (18%)	0.009
Diabetes Mellitus	5414 (1%)	5732 (3%)	0.108	3926 (2%)	4222 (2%)	0.010
Beta Blockers/Related	12688 (3%)	11560 (5%)	0.132	8460 (4%)	8900 (4%)	0.011
Family History of Ischemic Heart Disease	2243 (0%)	2994 (1%)	0.094	1765 (1%)	1985 (1%)	0.011
Hypertensive Diseases	9439 (2%)	13329 (6%)	0.209	8569 (4%)	9211 (5%)	0.016
Adverse Socioeconomic & Psychosocial Factors	3781 (1%)	4742 (2%)	0.113	2927 (1%)	3393 (2%)	0.019
Other Coagulation Defects	690 (0%)	2013 (1%)	0.107	687 (0%)	929 (0%)	0.019
Antihypertensives	4661 (1%)	5260 (2%)	0.110	3172 (2%)	3711 (2%)	0.021
Asian	18140 (4%)	8327 (4%)	0.005	8873 (4%)	8000 (4%)	0.022
Overweight And Obesity	23781 (5%)	27826 (13%)	0.273	19044 (9%)	20576 (10%)	0.026
Antimigraine Agents	18104 (4%)	23241 (11%)	0.265	16209 (8%)	17797 (9%)	0.028
Unknown Race	41267 (9%)	23073 (11%)	0.058	18354 (9%)	22097 (11%)	0.062
Other Race	25613 (6%)	9466 (4%)	0.055	11790 (6%)	8972 (4%)	0.063
Not Hispanic or Latino	325928 (71%)	146843 (68%)	0.063	144392 (72%)	137066 (68%)	0.079
Hispanic or Latino	45355 (10%)	29198 (14%)	0.114	20900 (10%)	27506 (14%)	0.101
White	333182 (73%)	142707 (66%)	0.139	144103 (72%)	134054 (67%)	0.108
Black Or African American	36945 (8%)	29920 (14%)	0.188	17229 (9%)	27061 (13%)	0.157

Aside from data regarding age, and number of patients (N), the remaining rows are sorted by ascending post-matching standardized mean difference (SMD). Race and ethnicity were not matched and are reported solely for descriptive purposes. Other abbreviations: At least two prescriptions of combined oral contraceptives (COC2), intrauterine devices (IUDs), not applicable (NA), standard deviation (SD).

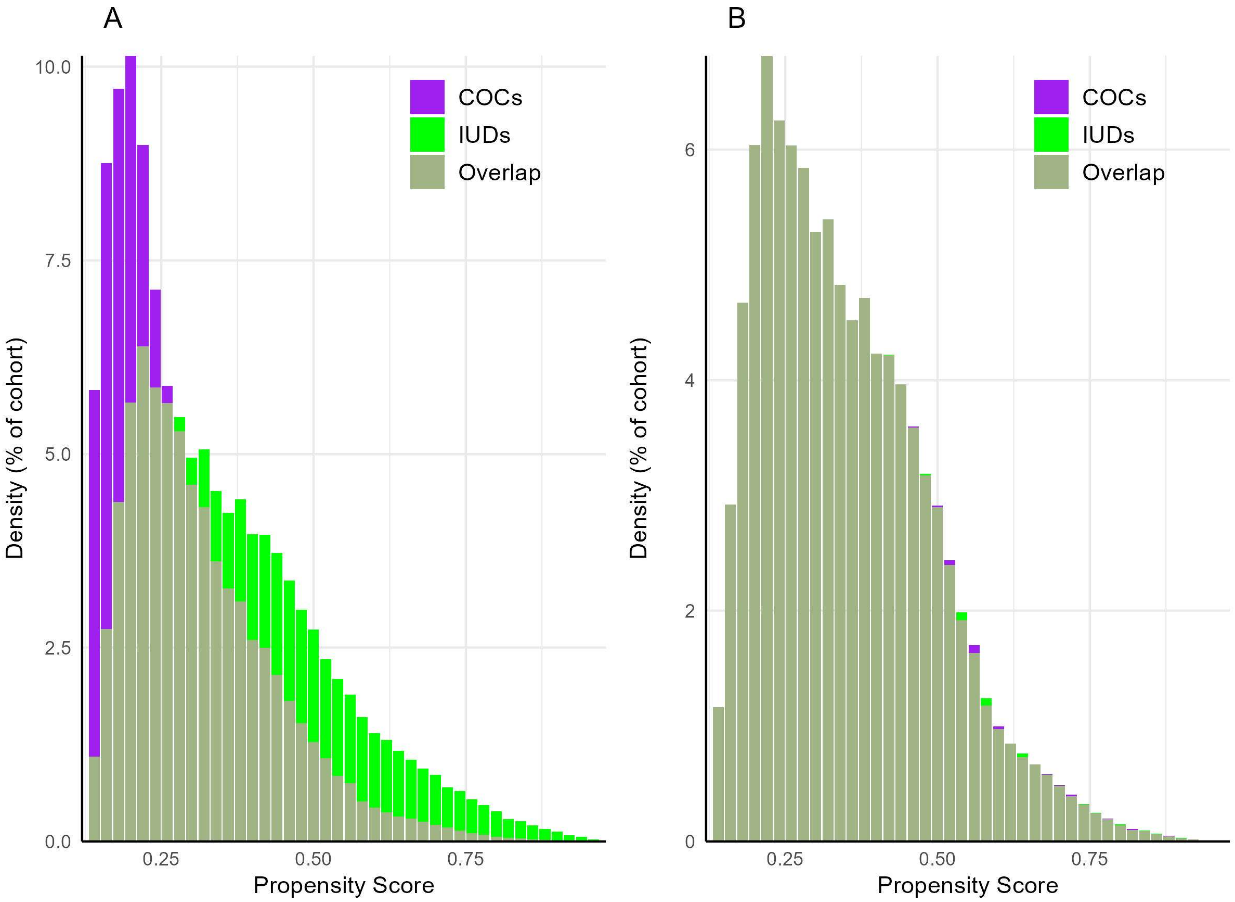

Figure 1: Propensity score density graph. Propensity scores before (A) and after (B) matching. The purple bars represent the combined oral contraceptives (COC2) cohort while the green bars represent the intrauterine devices (IUDs) cohort. Regions of propensity score density overlap, in which the shown proportion occurs in both cohorts, are shown by a darker shade of green. Following matching, propensity score densities overlap closely suggesting adequate covariate balance.

Key results

Similar to our main analysis, after matching, those prescribed at least two COCs (i.e., COC2 cohort) had an increase in risk of CeAD (RR=1.83 [1.03,3.26]; *P*=0.0357) and stroke (RR=1.78 [1.41,2.25]; *P*<0.0001) compared to the propensity-matched IUD cohort (Table 1 and Table 2).

Table 2: Outcomes for cervical artery dissection

	**Before matching**		**After matching***	
	COC2	IUDs	COC2	IUDs
Number of patients	457,386	214,766	201,372	201,372
CeAD n (%)	52 (0.011%)	19 (0.009%)	33 (0.016%)	18 (0.009%)
CeAD n per 100,000 person-years	10.5	8.8	16.4	8.9
CeAD risk ratio (95% CI)	1.29 (0.76,2.17); *P*=0.3482	Reference	1.83 (1.03,3.26); *P*=0.0357	Reference
Abbreviations: Cervical artery dissection (CeAD), at least two prescriptions of combined oral contraceptives (COC2), intrauterine devices (IUDs)<br>*Most relevant data for this sensitivity analysis				

Table 3: Outcomes for stroke

	**Before matching**		**After matching***	
	COC2	IUDs	COC2	IUDs
Number of patients	457,402	214,766	201,372	201,372
Stroke n (%)	310 (0.068%)	127 (0.059%)	194 (0.096%)	109 (0.054%)
Stroke n per 100,000 person-years	67.8	59.1	96.3	54.1
Stroke risk ratio (95% CI)	1.15 (0.93,1.41); *P*=0.1949	Reference	1.78 (1.41,2.25); *P*<0.0001	Reference
Abbreviations: Cervical artery dissection (CeAD), at least two prescriptions of combined oral contraceptives (COC2), intrauterine devices (IUDs)<br>*Most relevant data for this sensitivity analysis				

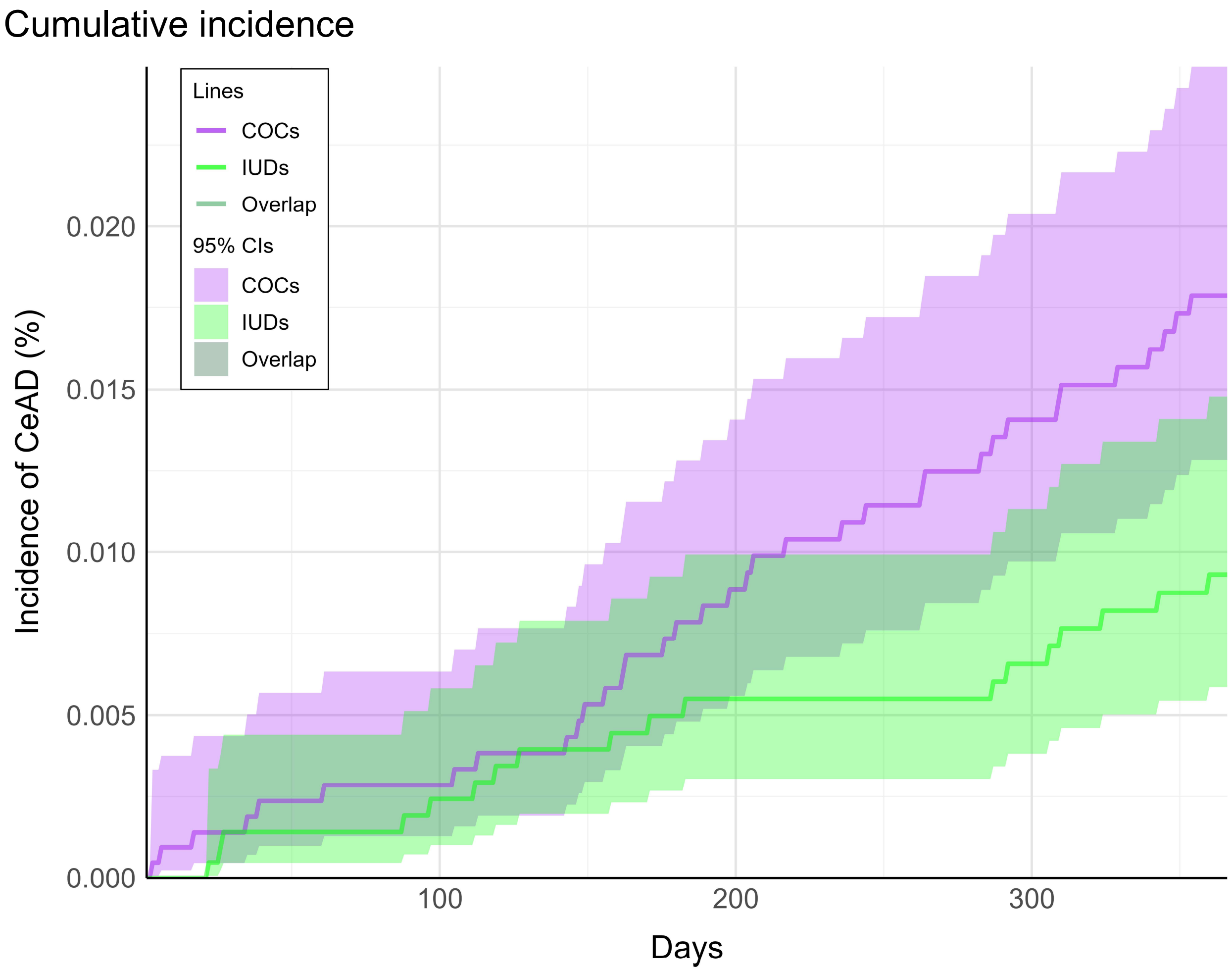

Figure 2: Cumulative incidence graph. Incidence curves for cervical artery dissection (CeAD) in the combined oral contraceptive cohort (COC2; purple lines) and intrauterine devices cohort (IUDs; green lines) are shown over the one-year follow-up period (365 days). Shaded ribbons indicate 95% confidence intervals. Regions in which the lines or 95% confidence intervals overlap between cohorts are shown in a darker shade of green.

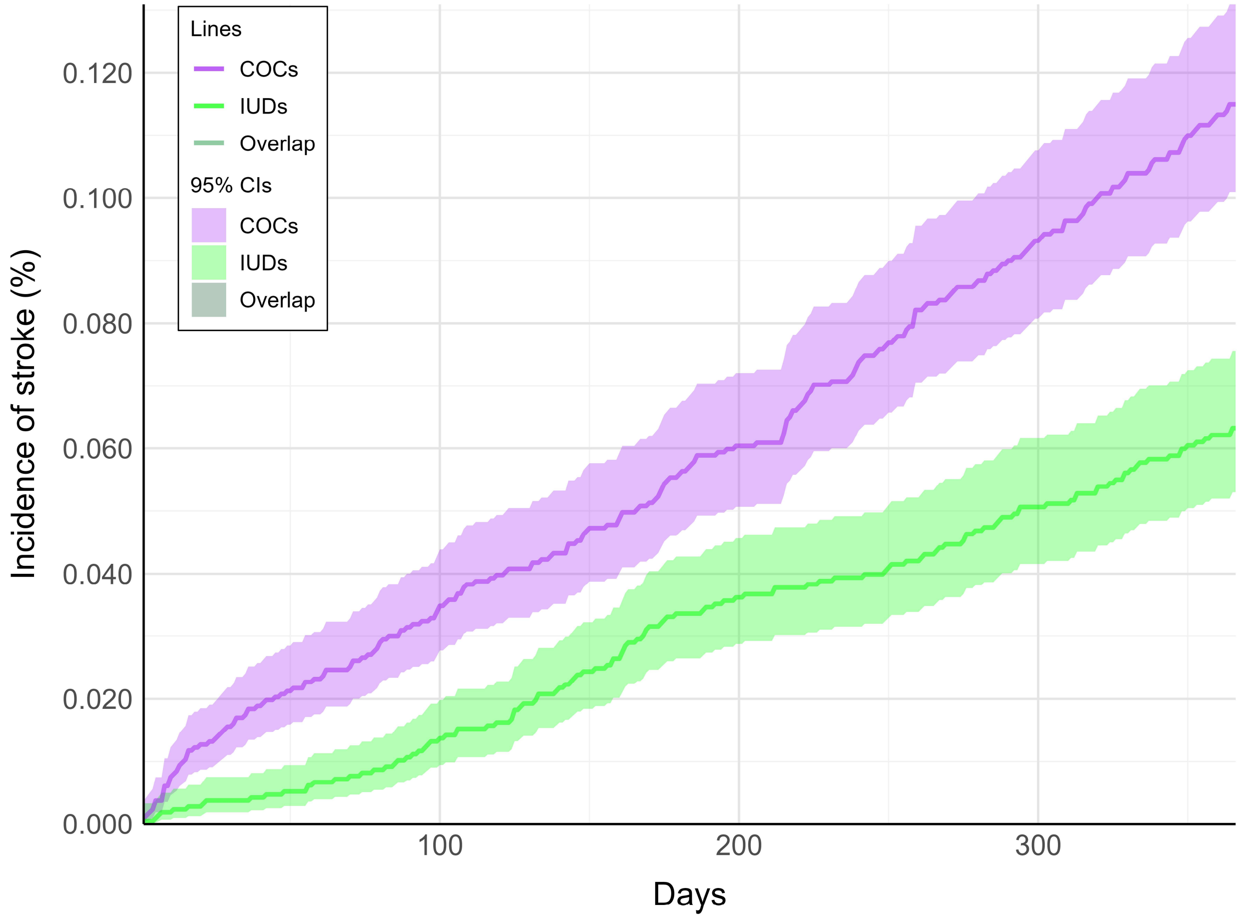

Figure 3: Cumulative incidence graph. Incidence curves for stroke in the combined oral contraceptive cohort (COC2; purple) and intrauterine devices cohort (IUDs; green) are shown over the one-year follow-up period (365 days). Shaded ribbons indicate 95% confidence intervals. Regions in which the lines or 95% confidence intervals overlap between cohorts are shown in a darker shade of green.

Negative control outcomes

- Format: COC2 vs. IUDs incidence, RR (95% confidence intervals)
  - Examination: 52% vs. 65%, RR=0.80 (0.80,0.81)
  - Human papillomavirus vaccine administration: 1.1% vs. 1.0%, RR=1.02 (0.96,1.09)
  - These outcomes further indicate balance between cohorts
